# Honokiol induces paraptosis*-*like cell death of acute promyelocytic leukemia via mTOR & MAPK signaling pathways activation

**DOI:** 10.1007/s10495-020-01655-9

**Published:** 2021-02-07

**Authors:** Xiaoli Liu, Yan Gu, Yaoyao Bian, Danhong Cai, Yu Li, Ye Zhao, Zhaofeng Zhang, Mei Xue, Liang Zhang

**Affiliations:** 1 Jiangsu Key Laboratory for Pharmacology and Safety Evaluation of Chinese Materia Medica, School of Pharmacy, Nanjing University of Chinese Medicine, Nanjing, Jiangsu 210023 People’s Republic of China; 2 Department of Geriatrics, The Second Hospital of Nanjing, Nanjing University of Chinese Medicine, Nanjing, Jiangsu 210003 People’s Republic of China; 3 College of Basic Medical Sciences, Institute of TCM-Related Comorbid Depression, Nanjing University of Chinese Medicine, Nanjing, Jiangsu 210023 People’s Republic of China

**Keywords:** Honokiol, Acute promyelocytic leukemia, Paraptosis, LC3, mTOR, MAPK

## Abstract

Acute promyelocytic leukemia (APL) is a blood system disease caused by the accumulation of a large number of immature blood cells in bone marrow. Although the introduction of all-*trans* retinoic acid (ATRA) and arsenic has reached a high level of complete remission rate and 5-year disease-free survival rate, the occurrence of various adverse reactions still severely affects the quality of life of patients. As a natural product, honokiol (HNK) has the advantages of low toxicity and high efficiency, and it is a potential drug for the treatment of cancer. Since cancer cells can escape apoptotic cell death through multiple adaptive mechanisms, HNK, a drug that induces cancer cell death in a nonapoptotic way, has attracted much interest. We found that HNK reduced the viability of human APL cell line (NB4 cells) by inducing paraptosis-like cell death. The process was accompanied by excessive reactive oxygen species (ROS), mitochondrial damage, endoplasmic reticulum stress, and increased microtubule-associated protein 1 light chain 3 (LC3) processing. The inactivation of proteasome activity was the main cause of misfolded and unfolded protein accumulation in endoplasmic reticulum, such as LC3II/I and p62. This phenomenon could be alleviated by adding cycloheximide (CHX), a protein synthesis inhibitor. We found that mTOR signaling pathway participated in paraptosis-like cell death induced by HNK in an autophagy-independent process. Moreover, the mitogen-activated protein kinase (MAPK) signaling pathway induced paraptosis of NB4 cells by promoting endoplasmic reticulum stress. In summary, these findings indicate that paraptosis may be a new way to treat APL, and provide novel insights into the potential mechanism of paraptosis-like cell death.

**Supplementary Information:**

The online version of this article (10.1007/s10495-020-01655-9) contains supplementary material, which is available to authorized users.

## Introduction

Acute promyelocytic leukemia (APL), belonging to the M3 subtype of acute myeloid leukemia, is characterized by the translocation of promyelocytic leukemia protein and retinoic acid receptor, leading to the formation of fusion proteins [1]. With the introduction of all-*trans* retinoic acid (ATRA), arsenic trioxide (ATO), and anthracyclines, the cure rate of APL has reached more than 90% [2]. However, some patients show resistance to ATRA and ATO [3, 4]. In addition, ATO and anthracyclines can damage the function of normal cells [3, 4]. Therefore, a nontoxic and effective natural medicine is needed.

Programmed cell death is divided into two types: caspase-dependent and caspase-independent. Caspase is closely related to cell apoptosis [5]. However, researchers have discovered that cancer cells could escape apoptosis through different mechanisms after a long struggle with cancer [6]. Therefore, caspase-independent cell death has gradually attracted researcher’s attention. Paraptosis is a new type of caspase-independent cell death [5], which is characterized by obvious vacuolization of the cytoplasm, swelling of endoplasmic reticulum and/or mitochondria, and endoplasmic reticulum stress [7, 8]. The swelling of the endoplasmic reticulum is caused by the accumulation of misfolded proteins and unfolded proteins, which is often mediated by mitogen-activated protein kinases (MAPKs), and inhibited by Alg-2 interacting protein X (Alix) [9].

In recent years, natural products such as curcumin [9] and morusin [10] have been widely studied due to low toxicity and effective anti-cancer activity. Honokiol (HNK) is a natural product extracted from Chinese herbal medicine Magnolia, which has many functions such as antioxidation, anti-inflammation, antibacterial, and antiviral [11]. The low toxicity of HNK to normal cells and its great anticancer potential have attracted much interest [12]. Previously, it has been found that HNK induced paraptosis of NB4 cells, but the underlying mechanism has not been elucidated in detail [13]. Here, we found that low-dose HNK induced paraptosis-like cell death in NB4 cells, but not apoptosis or cell cycle arrest. Our data suggested that paraptosis of NB4 cells was accompanied by excessive ROS, mitochondrial damage, and endoplasmic reticulum stress. Further mechanism studies have shown that HNK caused the accumulation of ubiquitinated proteins by inhibiting proteasome activity, leading to endoplasmic reticulum swelling. Surprisingly, we found that the upregulation of LC3II/I and p62 was related to paraptosis rather than autophagy. Finally, we verified that the paraptosis induced by HNK was closely related to mTOR and MAPK signaling pathways. These results indicate that HNK activates paraptosis by inducing mTOR and MAPK signaling pathways to promote vacuolation caused by endoplasmic reticulum stress.

## Materials and methods

### Cell culture and reagents

NB4 cells (BNCC341933) were purchased from BeNa Culture Collection (Beijing, China). The cells were cultured in RMPI 1640 medium (Gibco, Grand Island, NY, USA) containing 10% fetal calf serum (Gibco) and placed in a cell incubator at 37 °C and 5% CO2.

Honokiol and magnolol, purity ≥ 98%, were obtained from Purechem-Standard (Chengdu, Sichuan, China). Cycloheximide (CHX) (A8244), Z-VAD-FMK (A1902), rapamycin (A8167), 3-MA (A8353) and U0126 (A1337) were purchased from ApexBio (Houston, TX, USA). LY294002 (HY-10108), SP600125 (HY-12041) and SB203580 (HY-10256) were procured from MedChemExpress (Monmouth Junction, NJ, USA). (R)-MG132 (GC41233) was obtained from GLPBIO (Montclair, CA, USA). Suc-Leu-Leu-Val-Tyr-AMC (ab142120) was purchased from Abcam (Cambridge, MA, USA). Cleaved-caspase-3 (9661 T), JNK (9258P), p-JNK (Thr183/Tyr185) (9251P) and P38 (8690P) antibodies were purchased from Cell Signaling Technology (Danvers, MA, USA). Bax (50,599–2-lg), caspase-3 (19,677–1-AP), p21 (10,355–1-AP), p27 (25,614–1-AP), LC3 (14,600–1-AP), p62 (18,420- 1-AP), CHOP (60,304–1-lg) and β-actin (20,536–1-AP) antibodies were procured from Proteintech (Chicago, IL, USA). Bcl-2 (WL01556) and BiP (WL03157) antibodies were obtained from Wanleibio (Shenyang, Liaoning, China). Erk (AF0155), p-Erk (Thr202/Tyr204) (AF1015), p-P38 (Thr180/Tyr183) (AF4001), and ATF4 (DF6008) antibodies were purchased from Affinity Biosciences (Cincinnati, OH, USA). Ubiquitin (AF0306) antibody and SMER28 (SC5502) were obtained from Beyotime (Shanghai, China).

### Determination of cell viability

A cell counting kit-8 (CCK-8) was purchased from ApexBio (Houston). The cells were seeded in a 96-well plate at a density of 5*10^4^ cells/mL. Cells were treated with HNK for 24 h before performing the CCK-8 assay. Finally, the absorbance was measured at 450 nm using a microplate reader (TECAN, Männedorf, Switzerland).

### Optical microscope

NB4 cells were seeded at a density of 1 × 10^5^ cells/mL in a 24-well plate. Then, NB4 cells were treated with different concentrations of HNK and/or inhibitors for 24 h, and examined under an optical microscope (Nikon, Tokyo, Japan).

### Hoechst33258 staining

Apoptosis-Hoechst staining kit was obtained from Beyotime (Shanghai). The cells were seeded in a cell culture dish at 1 × 10^5^ cells/mL. Then, the cells were stained with Hoechst33258 staining solution for 5 min, and observed under a fluorescence microscope (Nikon).

### Cell cycle analysis

Cell Cycle Assay Kit was purchased from Fcmacs (NanJing, Jiangsu, China). The cells were collected, washed, fixed, stained with propidium iodide (PI) staining solution for 30 min, and then detected by flow cytometry (Biosciences Accuri C6, Franklin Lake, NJ, USA).

### Western blot

Cells were collected and lysed with the lysis buffer, containing protease and phosphatase inhibitors (RIPA: PMSF: protein phosphatase inhibitor = 100: 1: 1). A BCA protein concentration determination kit (Beyotime) was used to determine the protein concentration. The protein was separated with 8–12% SDS-PAGE and transferred to a nitrocellulose membrane (Millipore, Billerica, MA, USA). The membrane was blocked with TBST containing 5% skimmed milk for 1 h and incubated with primary antibodies overnight at 4 °C. Then, the membrane was washed with TBST, incubated with secondary antibody for 1 h, washed, and detected with a gel imager (Bio-Rad, Hercules, CA, USA).

### Immunofluorescence

NB4 cells were treated with different drugs, fixed and permeabilized with 0.1% Triton X-100 for 10 min. Next, the cells were blocked in 5% bovine serum albumin (BSA) for 30 min, and placed in ATF4 antibody (5% BSA: ATF4 = 100:1) overnight at 4 °C. After washing, the cells were incubated with a fluorescent secondary antibody for 2 h, counterstained with 4′,6-diamidino-2-phenylindole (DAPI) (Beyotime) for 10 min, and analyzed with a fluorescent microscope (Nikon).

### JC-1 staining

A mitochondrial membrane potential measurement kit (JC-1) (Beyotime) was used to detect the changes in mitochondrial membrane potential. The cells were collected by centrifugation, washed, and incubated with JC-1 staining working solution for 30 min at 37 °C. After washing, the cells were observed under a fluorescence microscope (Nikon).

### Detection of reactive oxygen species (ROS)

After discarding the cell supernatant, the cells were incubated in 10 µM 2′,7′-Dichlorodihydrofluorescein diacetate (DCFH-DA) (Beyotime) for 30 min. The cells were washed and detected with a fluorescence microscope (Nikon) or a fluorescence microplate reader (TECAN) with excitation and emission wavelengths of 488 and 525 nm.

### Fluorescent labeling of endoplasmic reticulum

According to the manufacturer’s instructions, endoplasmic reticulum-Tracker Red (Beyotime) was used to observe the morphological changes of endoplasmic reticulum. After fixation for 10 min, the cells were incubated in the endoplasmic reticulum-Tracker Red staining working solution for 30 min at 37 °C. Next, the cells were incubated with Hoechst3325 staining solution (Beyotime) for 5 min to stain the nucleus. Finally, the cells were analyzed under a fluorescence microscope (Nikon).

### 20S proteasome activity

After treating the cells with HNK for 24 h, the cells were collected, washed, and lysed with a lysis buffer without protease inhibitors. Then, the protein concentration was determined with BCA kit. In the presence or absence of (R)-MG-132(1 μM), the same amount of 30 μg protein, 20 μM fluorescent substrate (Suc-Leu-Leu-Val-Tyr-AMC) and a proper amount of buffer were distributed into a black 96-well plate with transparent substrate. After co-incubating at 37 °C for 1 h, the release of AMC (excitation 355 nm, emission 460 nm) was detected with a fluorescence microplate reader (TECAN).

### Real-time PCR

In a non-RNase environment, TRIzol reagent (Invitrogen, Carlsbad, CA, USA) was used to extract cellular RNA according to the manufacturer’s instructions. Then, the RNA was reverse transcribed using a 5X All-In-One RT MasterMix reverse transcription kit (Invitrogen, Carlsbad, CA, USA). The cDNA was amplified using EvaGreen 2X qPCR MasterMix reagent. GAPDH was used as an internal reference, and the expression of each target gene was calculated using the 2^−△△CT^ method. The primers used for amplification are as follows:

#### Alix

Primer F 5′-TCGCTGCTAAACATTACCAG-3’.

Primer R 5′-TGAGGGTCCCAACAGTATC-3’.

#### CHOP

Primer F 5′-CCTCACTCTCCAGATTCCAG-3’.

Primer R 5′-GCCACTTTCCTTTCATTCTC-3’.

#### ATF4

Primer F 5′-CCCTTCACCTTCTTACAACC-3’.

Primer R 5′-GAGGAGACCCCAGATAGGAC-3’.

#### BIP

Primer F 5′-TCCTATGTCGCCTTCACTC-3’.

Primer R 5′-ACAGACGGGTCATTCCAC-3’.

#### GAPDH

Primer F 5′-CACCATCTTCCAGGAGCGAG-3’.

Primer R 5′-AAATGAGCCCCAGCCTTCTC-3’.

### Statistical analysis

Each experiment was repeated at least three times, and all data were recorded as the mean ± SD. GraphPad Prism 6 software (graphpad prism, Lajolla CA, USA) was used for statistical analysis. T-test was used when comparing the differences between two groups, while a one-way analysis of variance (ANOVA) was used for the comparison between multiple groups. *P* < 0.05 was considered statistically significant.

## Results

### HNK can inhibit the viability of NB4 cells

**Fig. 1:**
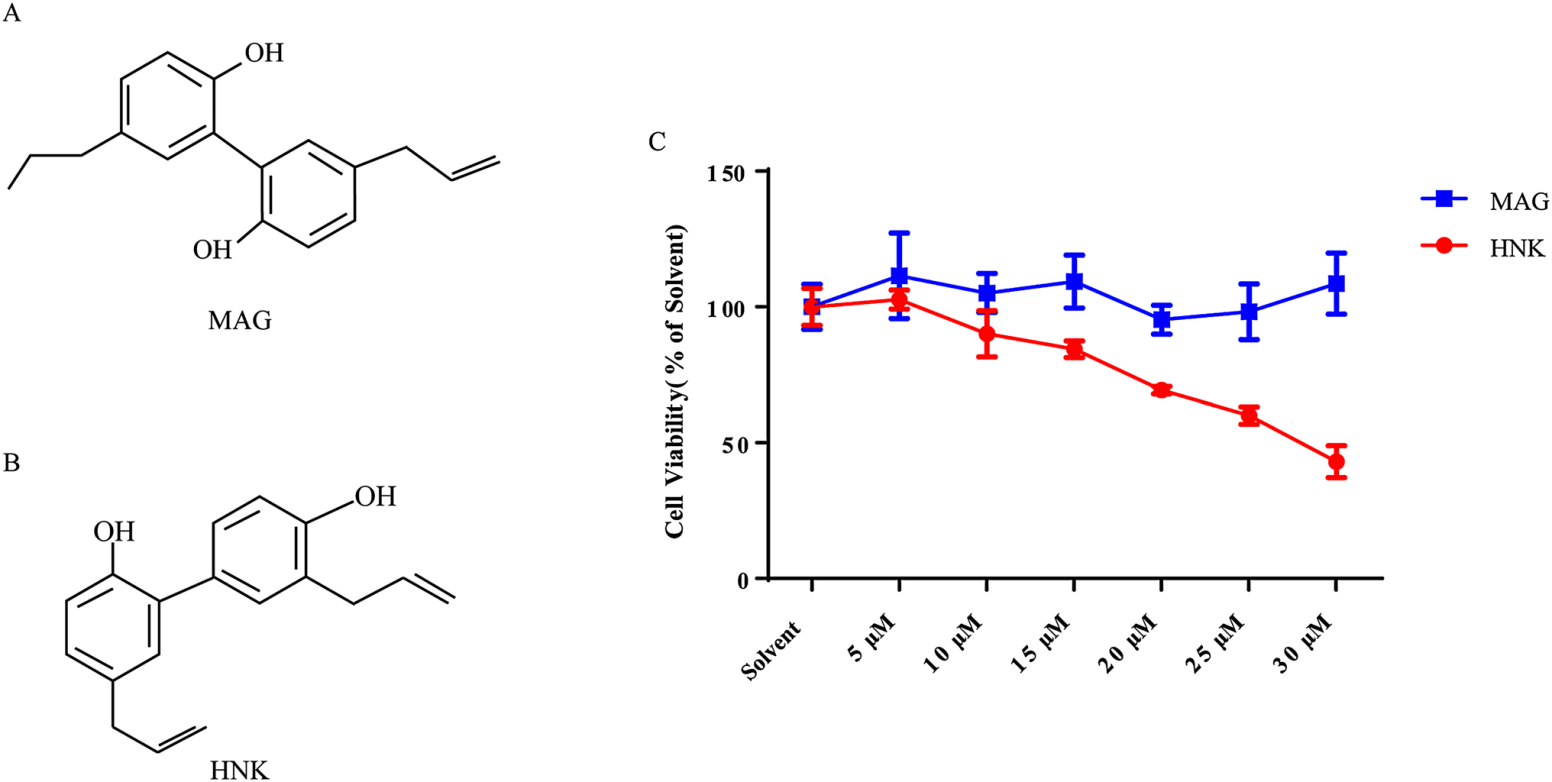
HNK reduces the viability of NB4 cells. **a** The chemical structure of MAG. **b** The chemical structure of HNK. **c** The effect of MAG and HNK on the viability of NB4 cells. NB4 cells were treated with different concentrations of MAG and HNK for 24 h, and cell viability was measured by CCK-8 assay. The results are expressed as mean ± SD (n = 6)

Magnolol (MAG) (Fig. 1a) and HNK (Fig. 1b) are the main biologically active components extracted from *Magnolia officinalis* (Magnoliaceae) [14]. The *M. officinalis* has high medicinal value, and different parts of medicine have different ingredients. Different doses of MAG and HNK were incubated with NB4 cells for 24 h. The sensitivity of NB4 cells to HNK was significantly higher than that of MAG (Fig. 1c).

### HNK induces paraptosis-like cell death in NB4 cells

**Fig. 2:**
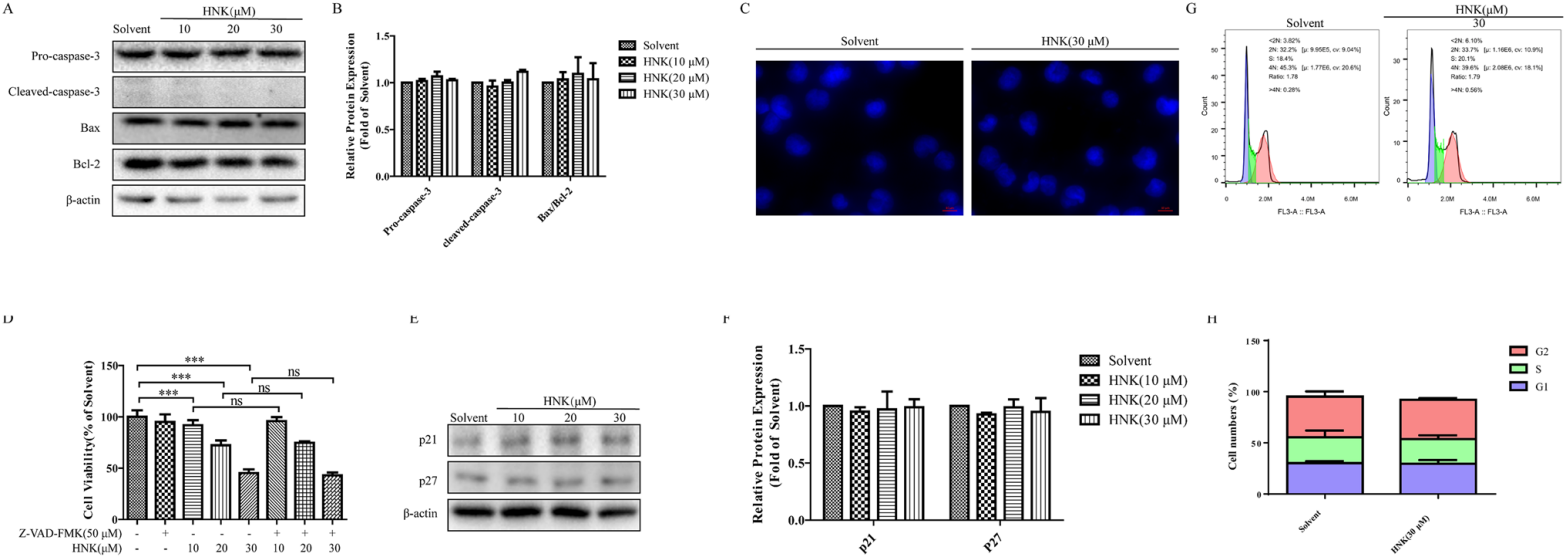
HNK cannot induce apoptosis and cell cycle arrest of NB4 cells. **a**, **b** NB4 cells were treated with HNK (0–30 μM) for 24 h, and the protein expression levels of Pro-caspase-3, Cleaved-caspase-3, Bax, and Bcl-2 were detected by western blot assay. β-actin was used as a loading control. The results are expressed as mean ± SD (n = 3). **c** Hoechst33258 staining was used to observe the morphological changes of NB4 cells after HNK (30 μM) incubation for 24 h. **d** NB4 cells were pretreated with Z-VAD-FMK for 2 h and then exposed to HNK (0–30 μM) for 24 h. The cell survival rate of each group was determined by CCK-8 assay. The results are expressed as mean ± SD (n = 6). Compared with solvent group, ***P < 0.001. Compared with the HNK group with a specified dose, ns: no significant difference. **e**, **f** Western blot analysis was used to detect the changes of cell cycle related proteins p21 and p27. The NB4 cells were treated with HNK (30 μM) for 24 h. **g**, **h** PI staining was used to detect the cell cycle distribution. The result is expressed as mean ± SD (n = 3)

Next, we explored the ways of NB4 cell death induced by HNK. Western blot results demonstrated that the expression of apoptosis proteins Pro-caspase-3, Cleaved-caspase-3, Bax, and Bcl-2 did not change significantly (Fig. 2a and b). Hoechst 33,258 staining did not show the fragmentation and condensation of cell nuclei (Fig. 2c). Z-VAD-FMK, a caspase inhibitor, is often used as an antiapoptotic drug [15, 16]. The addition of Z-VAD-FMK did not reverse the cell death induced by HNK (Fig. 2d). Together these results confirm that 30 μM HNK treatment of NB4 cells for 24 h does not cause apoptosis. Next, cell cycle arrest was detected by flow cytometry and western blotting in order to investigate whether the decrease of NB4 cell activity is related to cell cycle regulation. Interestingly, western blotting showed that 30 μM HNK did not cause cell cycle arrest (Fig. 2e–f), and flow cytometry also confirmed this result (Fig. 2g and h).

**Fig. 3:**
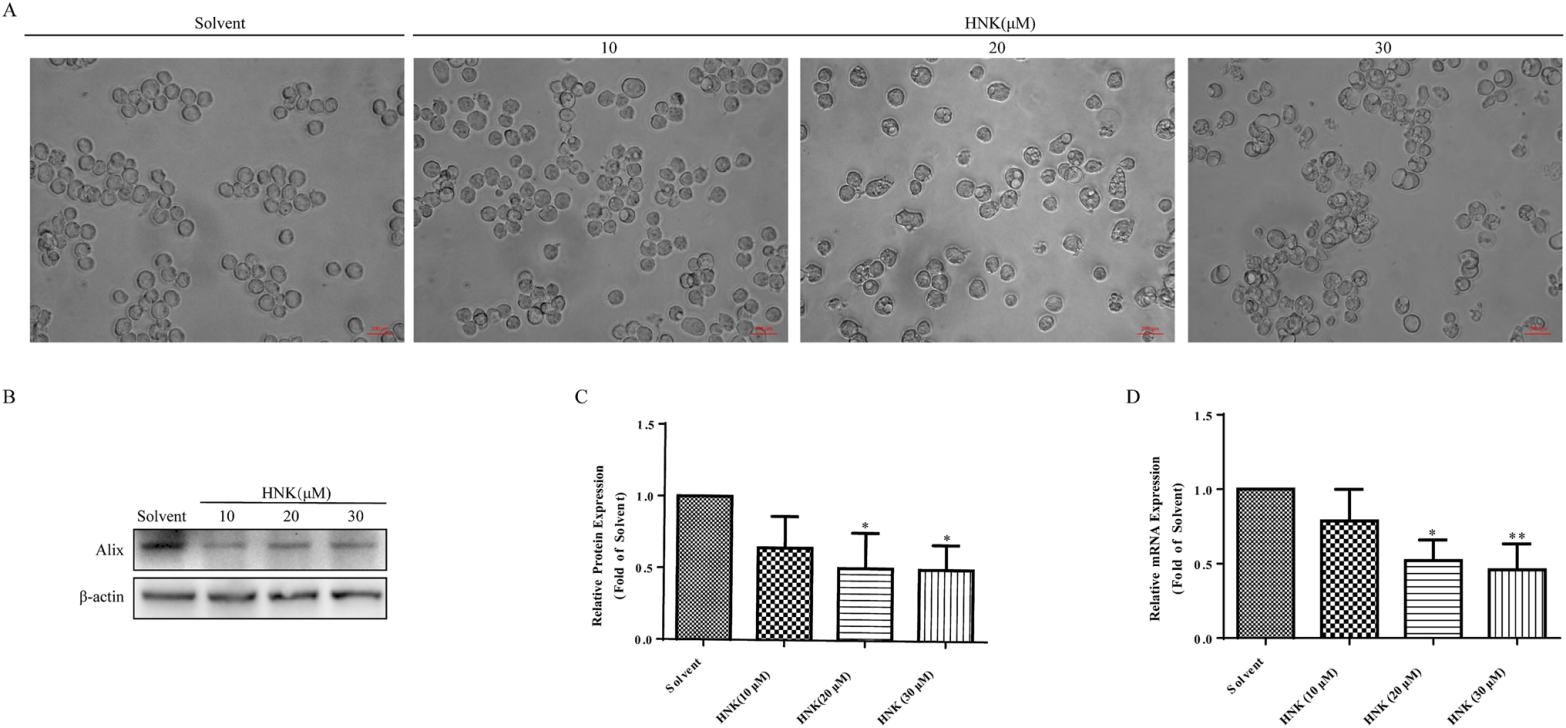
HNK induces the paraptosis of NB4 cells. The NB4 cells were incubated with 0, 10, 20, and 30 μM HNK for 24 h. **a** The morphological changes of cells were observed under an optical microscope. **b**, **c** The Alix protein expression was analyzed by western blotting. **d** Real-Time PCR was performed to measure the mRNA level of *Alix*. The results are expressed as mean ± SD (n = 3). Compared with the solvent group, *P < 0.05, **P < 0.01

Interestingly, optical microscopy showed that HNK induced extensive cytoplasmic vacuolation (Fig. 3a). The unique feature of paraptosis is vacuolation of the cytoplasm, mostly caused by swelling of the endoplasmic reticulum and/or mitochondria. In addition, paraptosis does not involve the activation of caspase, nucleus fragmentation and other apoptotic morphological characteristics [17]. Therefore, we hypothesized that HNK induced paraptosis-like cell death in NB4 cells. To confirm the above hypothesis, the expression level of Alix was tested. Western blotting and Real-Time PCR showed that the addition of HNK reduced the expression of Alix protein and mRNA (Fig. 3b–d), confirming that HNK induced paraptosis in NB4 cells.

### HNK-induced paraptosis of NB4 cells is accompanied by increased expression of ROS, mitochondrial damage, and endoplasmic reticulum stress

**Fig. 4:**
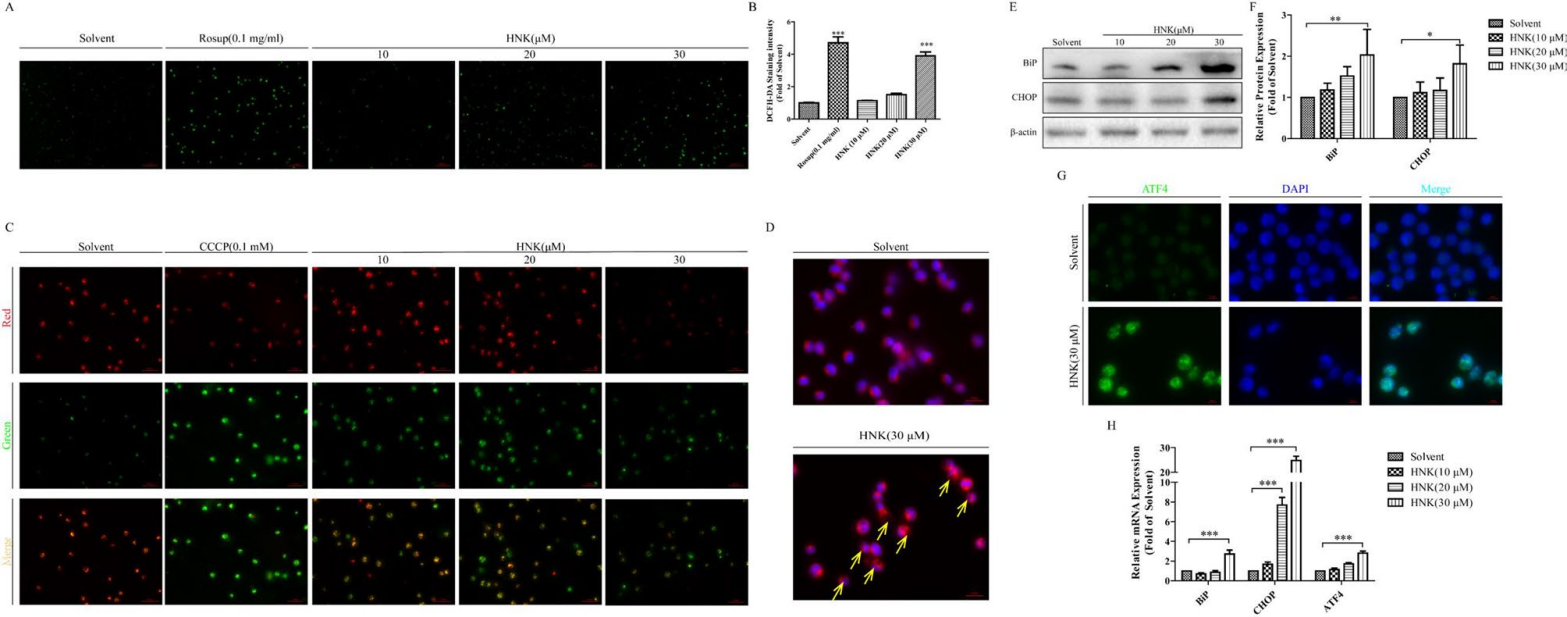
HNK can stimulate the mitochondrial damage and endoplasmic reticulum stress. The NB4 cells were incubated with 0, 10, 20, and 30 μM HNK for 24 h. **a** A fluorescent probe DCFH-DA detected the expression level of ROS in NB4 cells. 0.1 mg/mL Rosup was used as a positive control. **b** Measured the relative fluorescence intensity of each group of samples using a microplate reader. **c** Mitochondrial membrane potential was analyzed by JC-1. 0.1 mM CCCP was used as a positive control. **d** A specific endoplasmic reticulum fluorescent probe (red) was used to observe the formation of endoplasmic reticulum vacuoles (yellow allows) after 30 μM HNK treatment. **e**, **f** The endoplasmic reticulum stress protein BiP and CHOP were analyzed by western blotting. **g** The fluorescence intensity of endoplasmic reticulum stress protein ATF4 (green) was observed by immunofluorescence. **h** Real-Time PCR was used to measure the mRNA expression of *BiP*, *CHOP*, and *ATF4*. The results are expressed as mean ± SD (n = 3). Compared with the solvent group, *P < 0.05, **P < 0.01, and ***P < 0.001 (Color figure online)

Further experimental results showed that HNK induced a large amount of ROS production (Fig. 4a and b) and mitochondrial damage (Fig. 4c). It was previously reported that cytoplasmic vacuolation was mainly caused by endoplasmic reticulum swelling [18, 19]. Endoplasmic reticulum-specific labeling fluorescence confirmed that the cytoplasmic vacuolation induced by HNK was indeed caused by endoplasmic reticulum swelling (Fig. 4d). Western blotting and immunofluorescence confirmed that the expression of endoplasmic reticulum stress related proteins (BiP, CHOP, and ATF4) were significantly increased (Fig. 4e–g). Real-Time PCR was used to further investigate the endoplasmic reticulum stress mechanism of HNK. Our results showed that the *BiP, CHOP*, and *ATF4* mRNA levels were also increased after HNK treatment (Fig. 4h). These results confirm that the process of HNK-induced paraptosis is accompanied by increased expression of ROS, mitochondrial damage, and endoplasmic reticulum stress, and endoplasmic reticulum expansion is the main cause of cytoplasmic vacuolation.

### Proteasomal dysfunction contribute to HNK-induced paraptosis

**Fig. 5:**
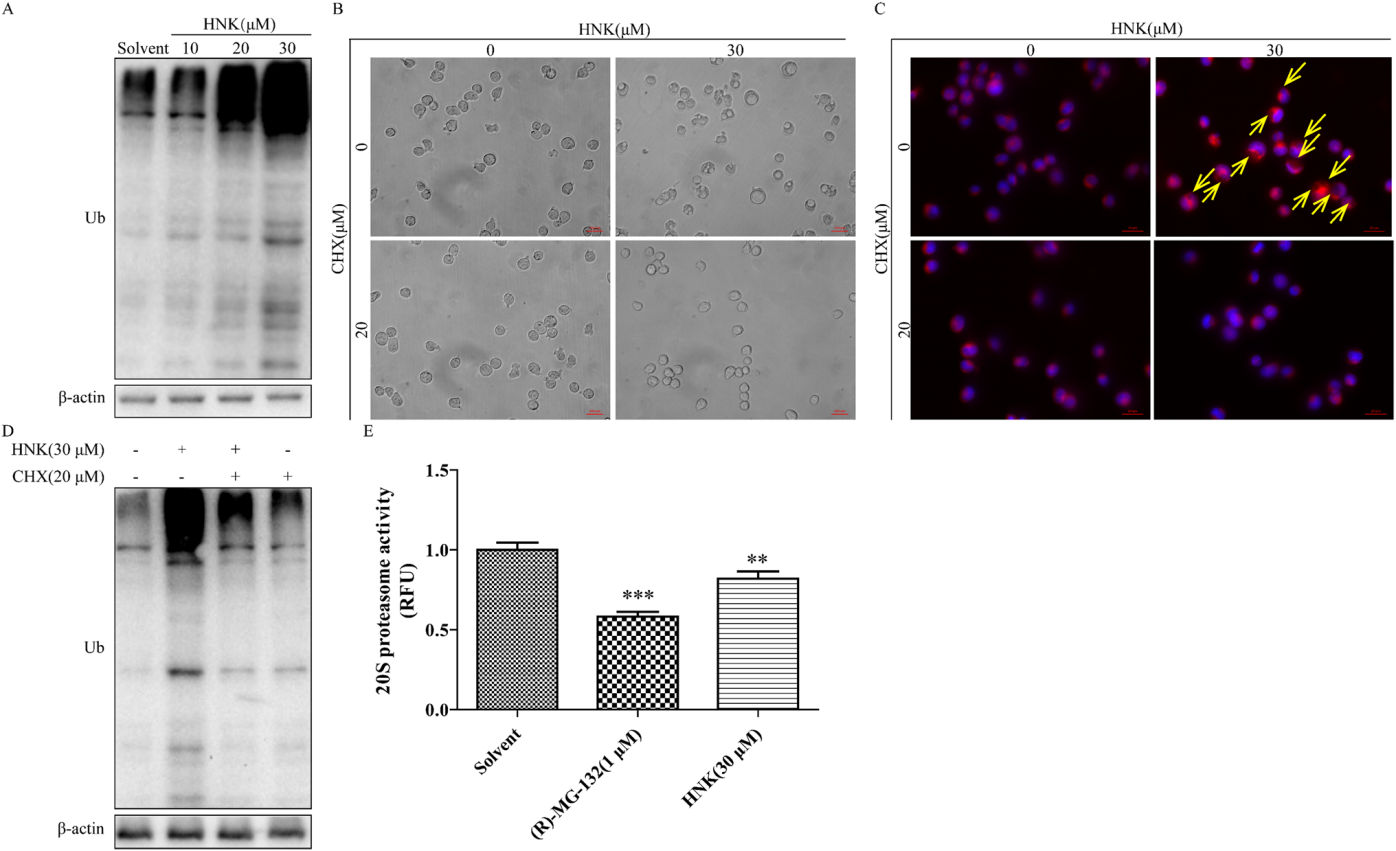
HNK increases protein ubiquitination by inhibiting proteasome activity. **a** The expression of ubiquitinated proteins after HNK treatment for 24 h. NB4 cells were pretreated with 20 μM CHX for 2 h and then incubated with HNK (0 or 30 μM) for 24 h. **b** Observed cytoplasmic vacuoles with a microscope. **c** Endoplasmic reticulum vacuoles (yellow allows) were marked using a specific endoplasmic reticulum fluorescent probe (red). **d** Western Blot analysis was used to measure the level of protein ubiquitination. **e** NB4 cells were treated with HNK (0 or 30 μM) for 24 h, and the cells were collected for 20S protease activity determination. 1 μM (R)-MG-132 was used as a positive control. The results are expressed as mean ± SD (n = 3). Compared with the solvent group, **P < 0.01 and ***P < 0.001 (Color figure online)

Expansion of endoplasmic reticulum is caused by misfolded or unfolded proteins accumulated in the endoplasmic reticulum [20]. Therefore, we need to determine the ubiquitination level of total protein in NB4 cells. Our experiments showed that HNK treatment increased the amount of ubiquitinated proteins (Fig. 5a). Next, cycloheximide (CHX) was added to evaluate the effect of protein synthesis inhibition on paraptosis [20]. The results showed that treatment with CHX for 2 h could prevent the HNK-induced endoplasmic reticulum vacuolation in NB4 cells (Fig. 5b and c). Furthermore, the addition of CHX also ameliorated the ubiquitination level of total protein caused by HNK (Fig. 5d). The accumulation of ubiquitinated proteins indicated that HNK can promote protein synthesis or inhibit proteasome activity. Next, the experimental data showed that HNK significantly inhibited the activity of proteasome (Fig. 5e). These results indicate that HNK can increase misfolded protein and unfolded protein by inhibiting proteasome activity, thereby causing endoplasmic reticulum swelling. CHX alleviates the paraptosis of cells by inhibiting protein synthesis.

### HNK-induced LC3 processing is paraptosis-dependent rather than autophagy

**Fig. 6:**
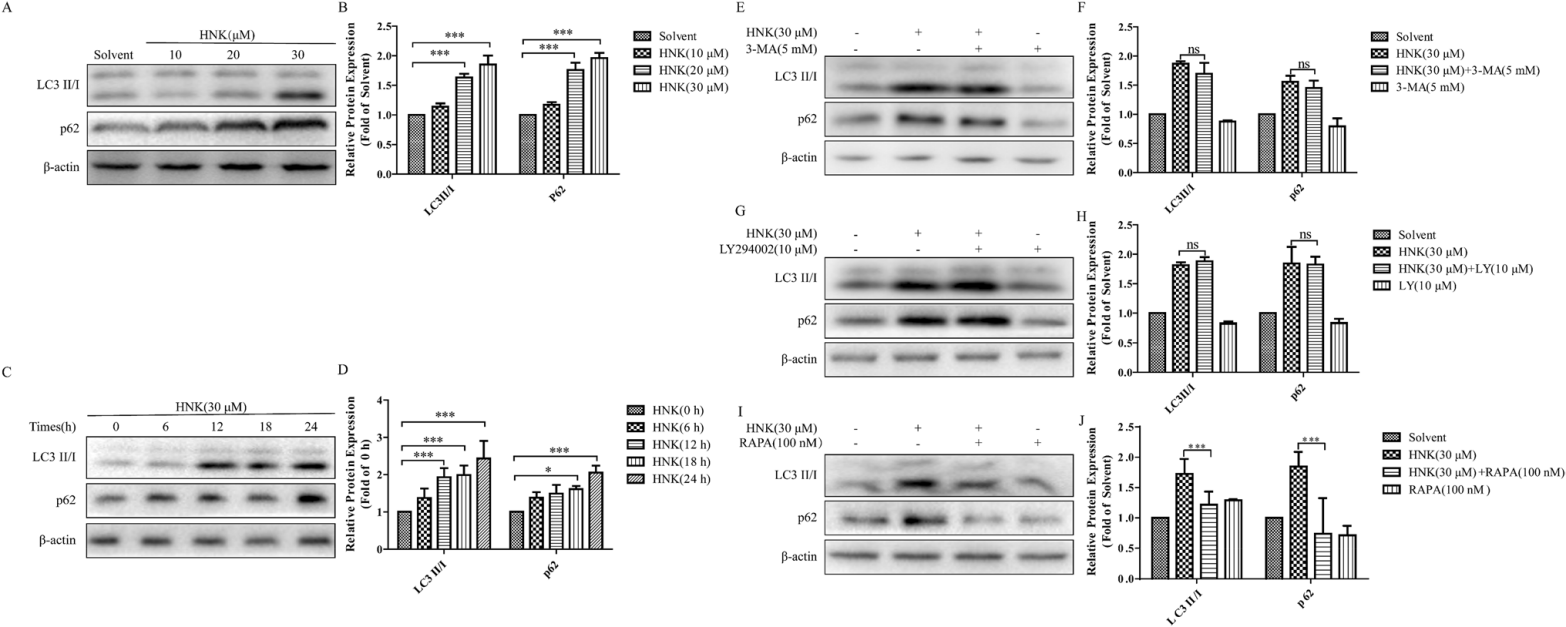
HNK can induce LC3 processing in an autophagy-independent manner. **a**, **b** NB4 cells were incubated with HNK (0–30 μM) for 24 h, and the expression of autophagy-related proteins LC3 II/I and p62 were detected. **c**, **d** Observed the expression levels of LC3 II/I and p62 protein after NB4 cells were stimulated with HNK (30 μM) for different times. The cells were divided into four groups: Solvent, HNK (30 μM), HNK + 3-MA/LY294002/rapamycin, and 3-MA/LY294002/rapamycin. **e**–**j** Expression of LC3 II/I and p62 proteins was detected by western blotting. The results are expressed as mean ± SD (n = 3). Compared with solvent group, *P < 0.05 and ***P < 0.001

**Fig. 7:**
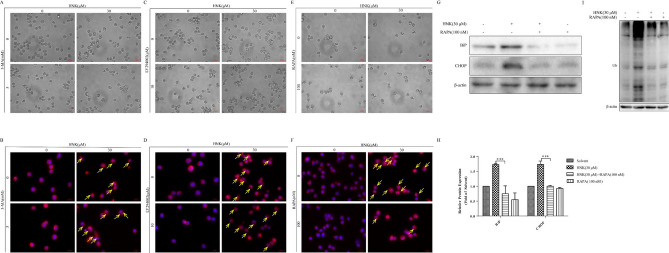
Inhibition of mTOR pathway can alleviate the generation of vacuolation. As shown in Fig. 6, the cells were divided into four groups. The cytoplasmic vacuolation (**a**, **c**, **e**) and endoplasmic reticulum expansion (yellow allows) (**b**, **d**, **f**) were observed under a microscope. **g**, **h** Endoplasmic reticulum stress proteins BiP and CHOP were analyzed by western blotting. **i** Western Blot analysis was used to measure the level of protein ubiquitination. The results are expressed as mean ± SD (n = 3). Compared with solvent group, ***P < 0.001

Our experiments proved that, inconsistent with only increasing the expression of LC3II/I during autophagy [21], the expression of LC3 II/I and p62 significantly increased after HNK treatment for 24 h (Fig. 6a-d). However, this phenomenon is probably caused by the instantaneous activation of autophagy and subsequent inhibition of autophagy [22]. Next, we explored whether HNK could induce the activation of early autophagy. It was found that a short-term treatment of HNK did not reduce the expression of p62, while the expression of LC3 II/I showed continuous increase (Fig. 6c and d). Therefore, we think that the processing of LC3 and increase of p62 expression are independent of autophagy. To more rigorously prove the experimental results, autophagy inhibitor (3-MA, LY294002) and autophagy activator (rapamycin, SMER28) were added. The addition of 3-MA and LY294002 did not change the expression of LC3 II/I and p62 induced by HNK (Fig. 6e–h). Moreover, 3-MA and LY294002 also did not affect endoplasmic reticulum vacuolization (Fig. 7a–d). The mTOR independent small molecule enhancer of autophagy, SMER28 [23–25], did not affect the expression of LC3II/I and p62 induced by HNK (Supplementary Fig. 1a and b), indicating that autophagy cannot participate in the death process of NB4 cell induced by HNK. Interestingly, rapamycin, could inhibit the increase of LC3II/I and p62 induced by HNK (Fig. 6i and j). This result is different from the autophagy-induced LC3 processing and p62 degradation mechanism [26, 27]. In autophagy, rapamycin activates autophagy by inhibiting the mTOR signaling pathway, which further promotes the increase of LC3II/I and the degradation of p62 [26, 27]. Overall, these results confirm that the increased expression of LC3II/I and p62 induced by HNK is autophagy-independent, which may be a unique phenomenon in paraptosis [20, 28]. Rapamycin may regulate the expression of paraptosis-related proteins LC3II/I and p62 by inhibiting the mTOR pathway.

### HNK-induced paraptosis of NB4 cells is related to mTOR pathway

In order to confirm the above hypothesis, we carried out further experimental verification. It was noticed that rapamycin could significantly inhibit endoplasmic reticulum vacuolation induced by HNK (Fig. 7e and f). Moreover, western blot results revealed that the addition of rapamycin relieved endoplasmic reticulum stress (Fig. 7g and h). Notably, rapamycin also prevented HNK-induced the increase of protein ubiquitination (Fig. 7i). Rapamycin is the inhibitor of mTOR signaling pathway. Previous studies reported that inhibiting the short-term activation of the mTOR signaling pathway by NIM811 (a small-molecule cyclophilin binding inhibitor) significantly reduced the formation of vacuoles [22]. Therefore, the above evidences prove that rapamycin can reduce the accumulation of misfolded protein aggregates (such as LC3II/I and p62) by inhibiting mTOR signaling pathway, and regulate the vacuolation of endoplasmic reticulum in autophagy-independent manner.

### MAPK signaling pathway is involved in HNK-induced paraptosis

**Fig. 8:**
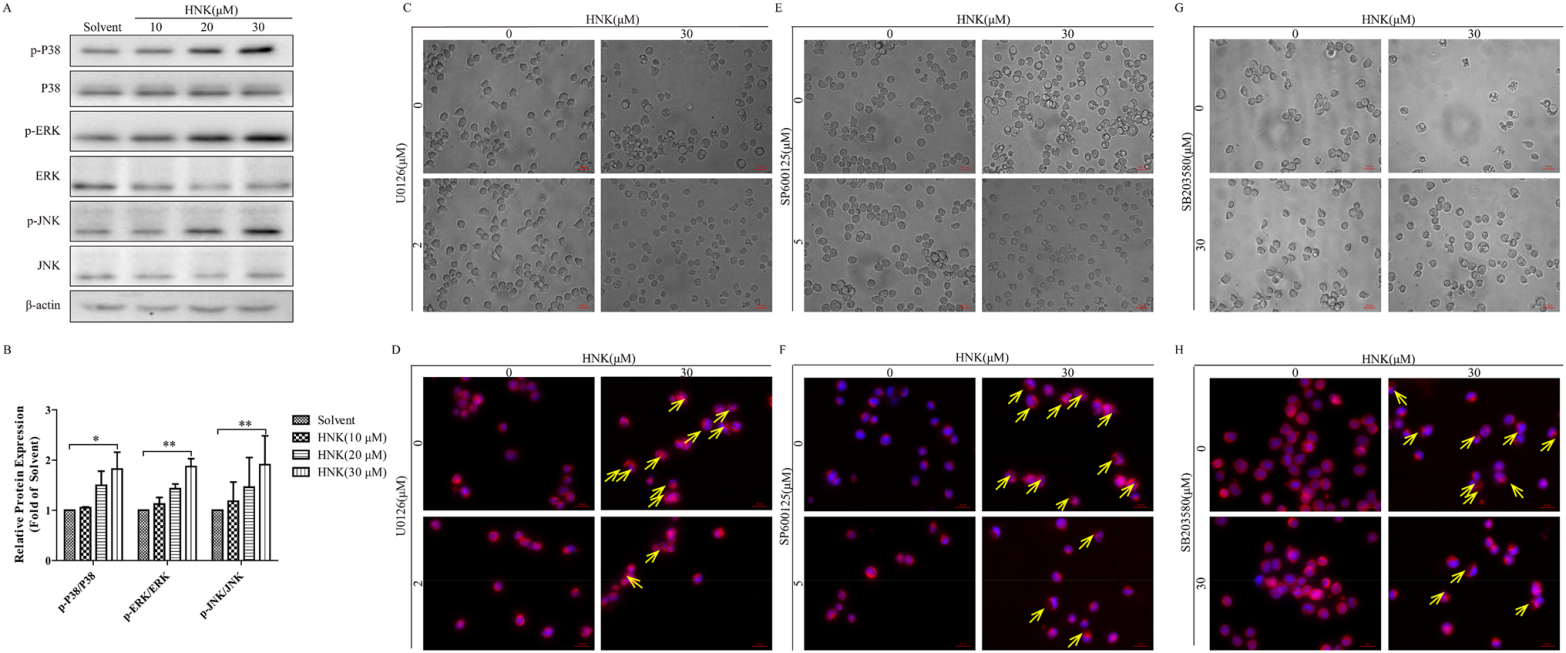
HNK can activate the MAPK signaling pathway. (**a**-**b**) NB4 cells were stimulated with 0, 10, 20, and 30 μM HNK for 24 h, and the expression of MAPK signaling pathway related proteins was determined. The cells were divided into four groups: Solvent, HNK (30 μM), HNK + U0126/SP600125/SB203580, U0126/SP600125/SB203580. The vacuolation of cytoplasm (**c**, **e**, **g**) and swelling of endoplasmic reticulum (yellow allows) (**d**, **f**, **h**) were observed. The results are expressed as mean ± SD (n = 3). Compared with the solvent group, *P < 0.05 and **P < 0.01

**Fig. 9:**
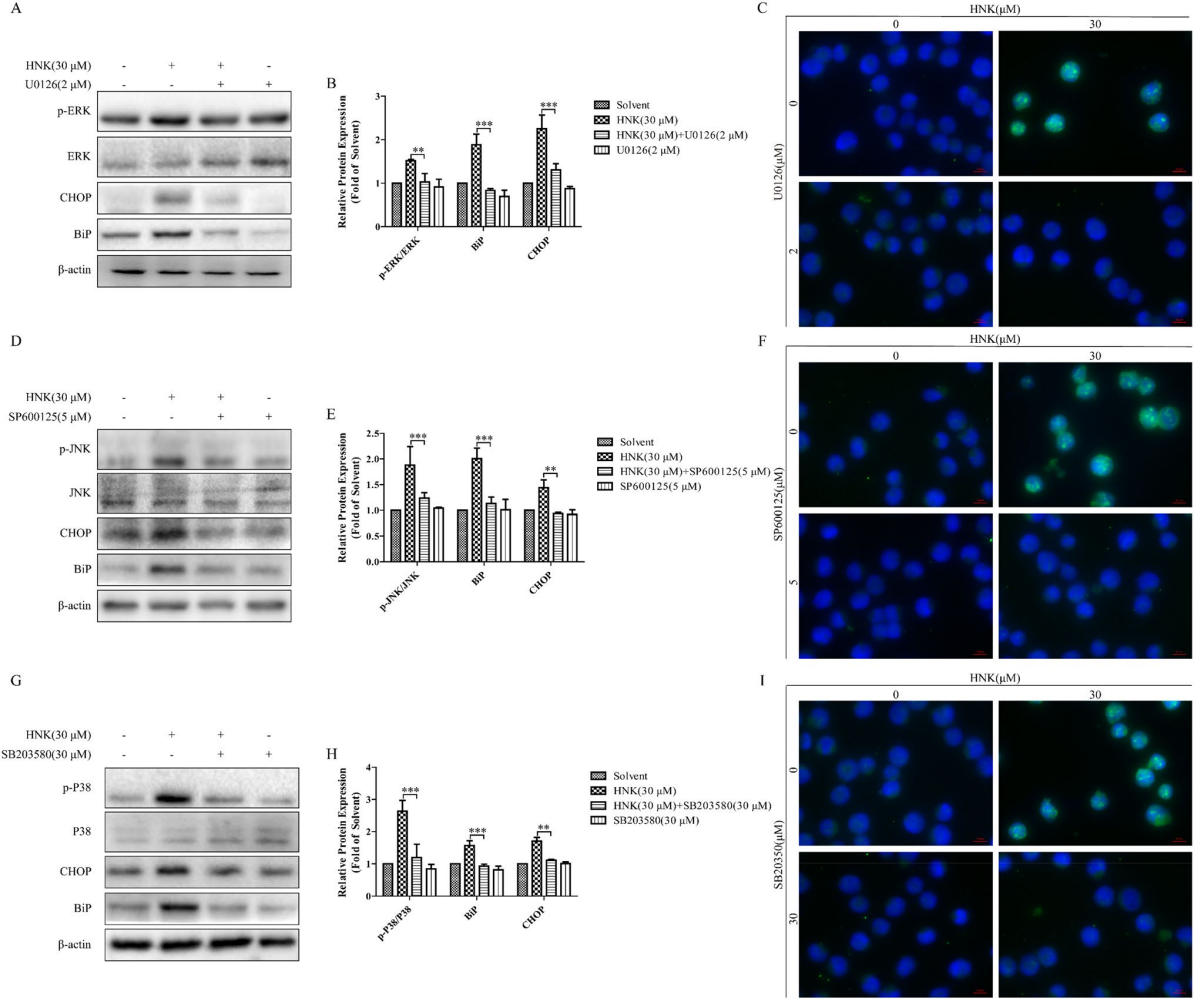
MAPK signaling pathway is involved in the HNK-induced endoplasmic reticulum stress. As shown in Fig. 8, the cells were divided into four groups. (**a**, **b**, **d**, **e**, **g**, **h**) MAPK signaling pathway proteins and endoplasmic reticulum stress proteins (BiP and CHOP) were analyzed by western blotting. (**c**, **f**, **i**) The expression of ATF4 was determined by immunofluorescence. The results are expressed as mean ± SD (n = 3). Compared with HNK (30 μM) group, **P < 0.01 and ***P < 0.001

To clarify the signal mechanisms of paraptosis induced by HNK, the activation of MAPK was analyzed by western blotting. After treatment with HNK, the expression levels of p-P38, p-ERK and p-JNK were significantly upregulated (Fig. 8a and b). Moreover, the addition of small-molecule inhibitors of MAPK pathway prevented HNK-induced endoplasmic reticulum vacuolation (Fig. 8c–h). Pretreatment of cells with three inhibitors could significantly relieve endoplasmic reticulum stress (Fig. 9a, b, d, e, g, h). Finally, decreased fluorescence intensity of ATF4 was observed in the three inhibitor pretreatment groups, indicating that endoplasmic reticulum stress was improved (Fig. 9c, f, i). These results confirm that MAPK signaling pathway is involved in the paraptosis induced by HNK.

## Discussion

**Fig. 10:**
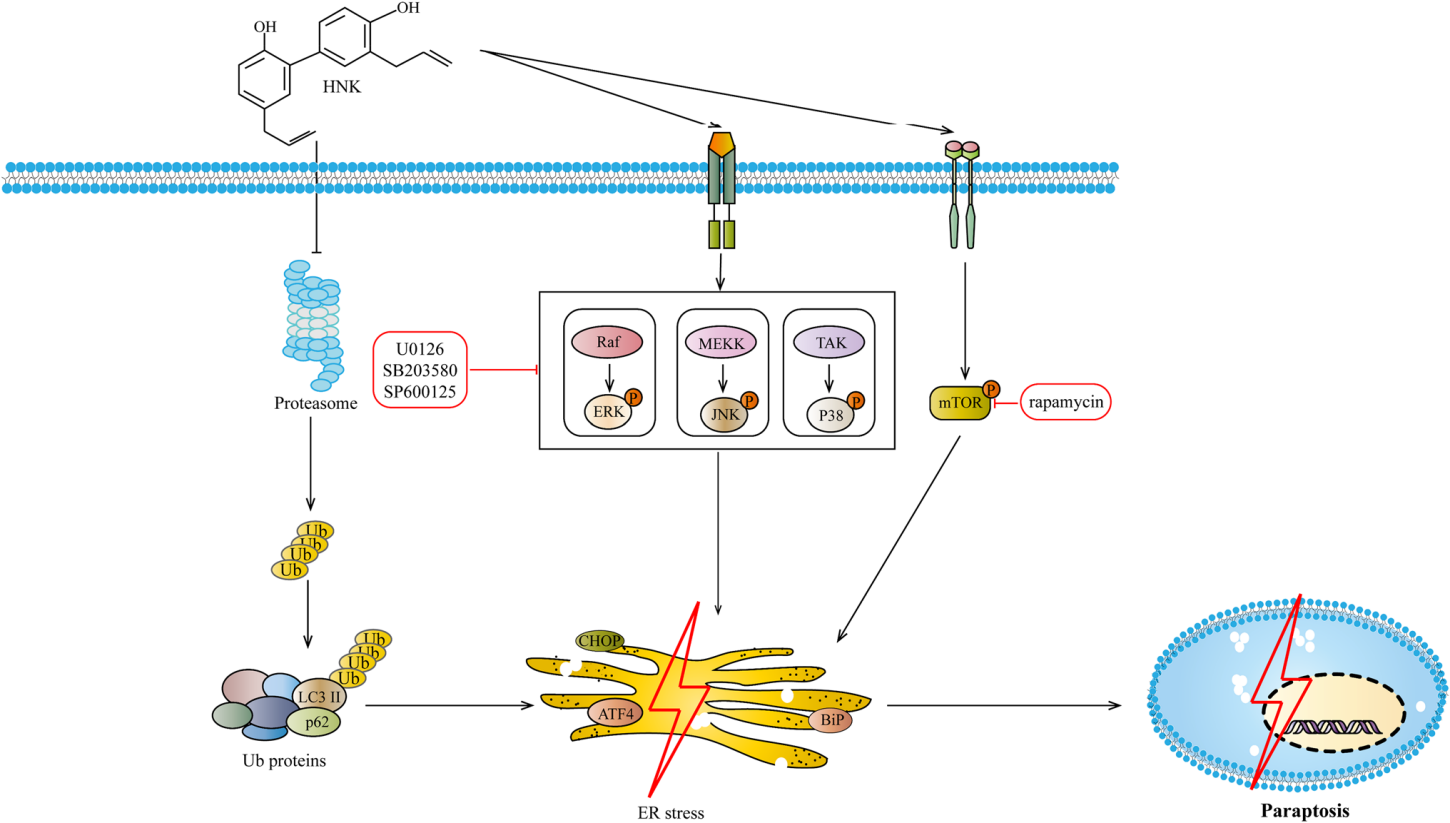
A schematic illustration of the mechanism of HNK inducing paraptosis-like cell death in NB4 cells

In recent years, HNK has gradually been recognized due to its significant anticancer activity and high pharmacological safety [12, 29]. Although the cure rate of APL has been significantly improved, the severe adverse reactions caused by ATRA and ATO have not been resolved [30]. Moreover, traditional anti-tumor drugs exert anti-tumor effect mainly through common cell death modes such as apoptosis, differentiation and so on [31, 32]. However, cancer cells have many against apoptosis and differentiation mechanisms to escape death, which were considered to be important reasons for deterioration, drug resistance and recurrence [31, 32]. Therefore, it is essential to explore and utilize new methods of cell death for cancer treatment [33, 34]. As a natural product, HNK has the advantages of low toxicvity and high efficiency, and is expected to become a potential drug for the treatment of cancer [11, 12]. In this context, we confirmed the molecular mechanism of HNK-induced paraptosis death of NB4 cells (Fig. 10).

First, it was found that HNK reduced the viability of NB4 cells (Fig. 1c). Interestingly, neither nucleus morphology nor apoptosis proteins was changed (Fig. 2a–d). Further experimental results showed that the cells did not undergo cycle arrest (Fig. 2e–h). These results suggest that HNK inhibit the viability of NB4 cells by nonapoptotic programmed death manner. Paraptosis is a new type of cell death accompanied by cytoplasmic vacuoles, unlike many well-known cell death modes. Cytoplasmic vacuolation suggested that HNK may induce paraptosis-like cell death in NB4 cells. Alix is a specific marker of paraptosis, and Alix can inhibit paraptosis but not apoptosis [35]. The decreased expression of Alix confirmed that paraptosis occurred in NB4 cells (Fig. 3). The levels of ROS are known to play a vital role in cancer treatment [36]. Our data revealed that HNK promoted the accumulation of ROS in NB4 cells, which is similar to other related reports of paraptosis [9, 37]. The abnormal increase of ROS not only changes the mitochondrial membrane potential [38, 39] but also affects the morphology of endoplasmic reticulum [9]. Here, it was found that HNK could stimulate the mitochondrial damage and endoplasmic reticulum stress. Moreover, endoplasmic reticulum expansion was the main cause of cytoplasmic vacuolation (Fig. 4).

What is the mechanism of HNK-induced endoplasmic reticulum expansion? To solve this problem, the ubiquitination level of total protein was checked. The results showed that HNK could trigger the accumulation of misfolded and unfolded proteins in the endoplasmic reticulum and expand the endoplasmic reticulum to accommodate these proteins, eventually leading to endoplasmic reticulum stress. The addition of CHX completely alleviated vacuolation and the production of ubiquitin protein (Fig. 5), and similar results were obtained in other paraptosis studies [20, 40]. The increase of ubiquitinated proteins indicates that HNK may promote protein synthesis or inhibit protein degradation. Application of proteasome activity assay kit showed that HNK achieved similar results as (R)-MG132, a proteasome inhibition (Fig. 5). The results show that HNK inhibits the activity of proteasome to reduce protein degradation, thereby accumulating proteins in the endoplasmic reticulum and causing the expansion of endoplasmic reticulum.

Next, it was found that HNK promoted the expression of LC3 II/I and p62 in a dose-dependent manner (Fig. 6a and b), which attracted our interest. There are two different explanations for this phenomenon. One is that drugs can induce the transient activation of autophagy, and the other is that the processing of LC3 and the upregulation of p62 are symptoms of paraptosis [22, 28]. Further results showed that the expression of p62 protein increased continuously, which confirmed that the instantaneous activation of autophagy did not occur. Moreover, the addition of autophagy inhibitors did not change HNK-mediated LC3 processing (Fig. 6e–h). SMER28, a mTOR-independent autophagy enhancer, also did not affect the expression of LC3II/I and p62 increased by HNK (Supplementary Fig. 1a and b). These results suggest that the anti-NB4 cells of HNK are independent of autophagic processes. Interestingly, rapamycin could inhibit the processing of LC3 and the accumulation of p62 induced by HNK, which is not a characteristic of autophagy. Rapamycin promotes the autophagy process by inhibiting the mTOR signaling pathway to further increase the expression of LC3II/I and accelerate the degradation of p62 [26, 27]. Therefore, rapamycin may be involved in the paraptosis process induced by HNK, thereby affecting the expression of LC3II/I and p62. These phenomena are common in paraptosis caused by other drugs including Manumycin A [28], and Plumbagin [41]. The processing of LC3 and the increase of p62 may be caused by the inhibition of proteasome activity. Next, we found that rapamycin prevented endoplasmic reticulum vacuolation and protein ubiquitination (including LC3 and p62), which confirmed our above hypothesis (Fig. 7). Previous studies reported that inhibition of mTOR signaling pathway could significantly ameliorate the formation of vacuoles [22]. These evidences suggest that rapamycin is involved in HNK-induced paraptosis by inhibiting the mTOR signaling pathway in an autophagy-independent manner. However, the specific mechanism of mTOR signaling pathway regulating HNK-induced paraptosis cell death is still unclear.

A large number of studies reported that the MAPK signaling pathway can participate in paraptosis-like cell death [9, 42, 43]. We hypothesized that HNK induced the paraptosis of NB4 cells through the MAPK signaling pathway. First, HNK could significantly promote the phosphorylation of P38, ERK, and JNK. Moreover, the addition of inhibitors of the MAPK signaling pathway could significantly prevent HNK-induced vacuolation (Fig. 8) and endoplasmic reticulum stress (Fig. 9). The results indicate that HNK can trigger the paraptosis of NB4 cells by activating the MAPK signaling pathway.

Overall, this study shows that HNK induces NB4 cells paraptosis via inhibiting the activity of proteasome. Inhibitors of mTOR and MAPK signaling pathway can alleviate the paraptosis induced by HNK. These results suggest that HNK is a potential drug for anti-APL.

## Supplementary Information

Below is the link to the electronic supplementary material.

Fig. S1 SMER268, a small molecule enhancer of autophagy, cannot affect the expression of LC3II/I and p62 induced by HNK. The cells were divided into four groups: Solvent, HNK (30 μM), HNK+SMER28, and SMER28 (50 μM). (A-B) Western blot analysis determined the LC3 II/I and p62 protein expression levels. The results are expressed as mean ±SD (n = 3) (pptx 575 kb)
