# Supplementary figures and images for: Honokiol induces paraptosis-like cell death of acute promyelocytic leukemia via mTOR & MAPK signaling pathways activation
Source: Apoptosis. 2021 Feb 7;26(3):195–208. doi: 10.1007/s10495-020-01655-9 (PMC8016806; doi:10.1007/s10495-020-01655-9)

## Slide 1
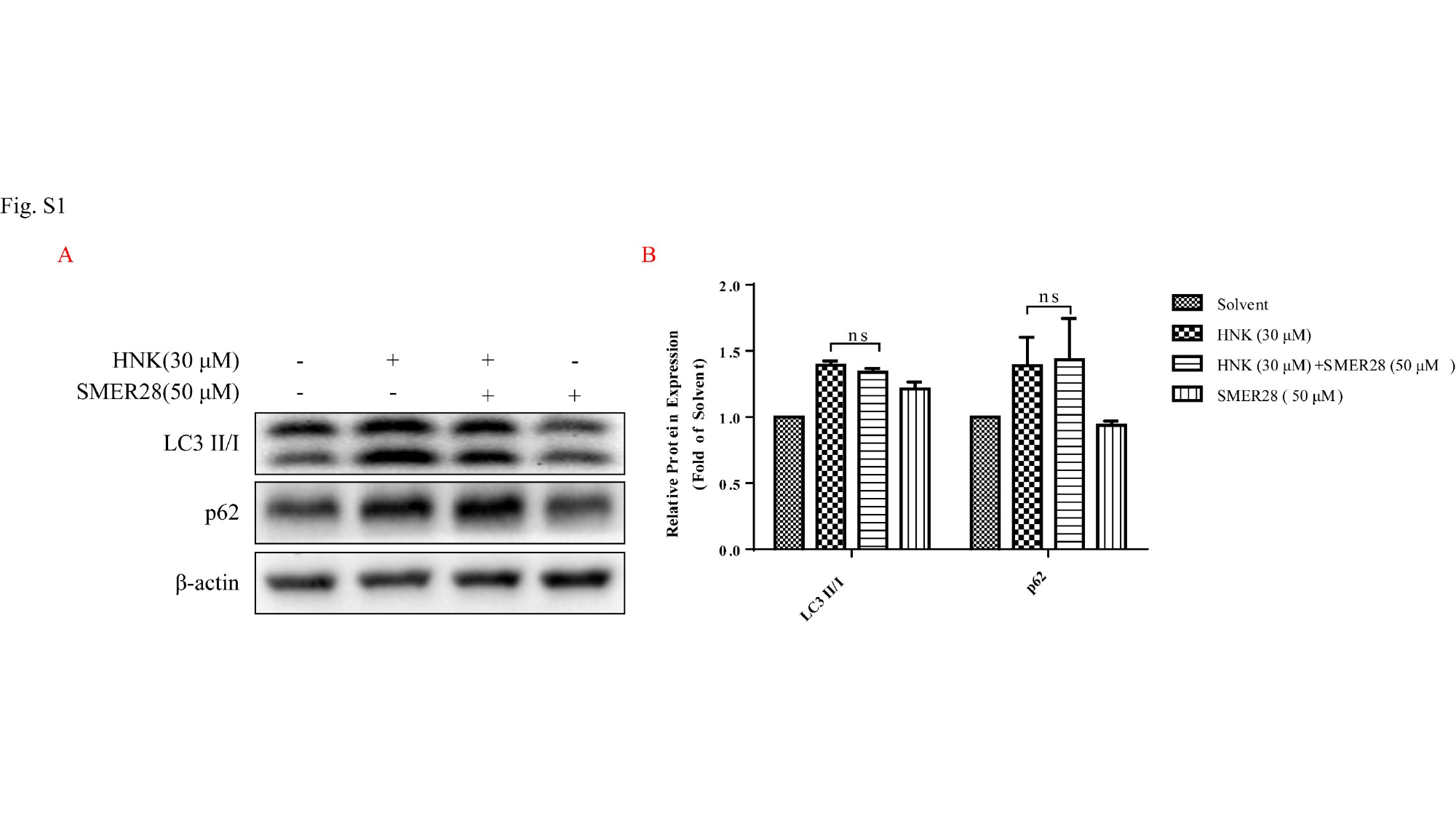

Supplement: Supplementary file 1 — Fig. S1 SMER268, a small molecule enhancer of autophagy, cannot affect the expression of LC3II/I and p62 induced by HNK. The cells were divided into four groups: Solvent, HNK (30 μM), HNK+SMER28, and SMER28 (50 μM). (A-B) Western blot analysis determined the LC3 II/I and p62 protein expression levels. The results are expressed as mean ±SD (n = 3) (pptx 575 kb) [file 10495_2020_1655_MOESM1_ESM.pptx]
